# A Systematic Review and Meta-Analysis of Clinical Outcomes of Intravitreal Anti-VEGF Agent Treatment Immediately after Cataract Surgery for Patients with Diabetic Retinopathy

**DOI:** 10.1155/2019/2648267

**Published:** 2019-04-16

**Authors:** Li-Quan Zhao, Jin-Wei Cheng

**Affiliations:** ^1^Department of Ophthalmology, Pudong New Area People's Hospital Affiliated to Shanghai University of Medicine & Health Sciences, Shanghai, China; ^2^Department of Ophthalmology, Shanghai General Hospital, Shanghai Jiao Tong University School of Medicine, Shanghai, China

## Abstract

**Aims:**

To examine possible benefits of intravitreal anti-vascular endothelial growth factor (VEGF) agent treatment immediately after cataract surgery for patients with diabetic retinopathy (DR).

**Methods:**

A comprehensive literature search was performed using the Cochrane collaboration methodology to identify randomized controlled trials (RCTs) and comparative studies of cataract surgery with or without anti-VEGF agent treatment for any diabetic retinopathy. Meta-analyses were performed for clinical outcome parameters including changes in macular thickness (MT), best-corrected visual acuity (BCVA), incidence of diabetic retinopathy and maculopathy progression, laser treatment rate, and other complications.

**Results:**

Nine RCTs and 3 nonrandomized comparative studies were identified and used for comparing cataract surgery with intravitreal bevacizumab (IVB) or intravitreal ranibizumab (IVR) treatment (338 eyes, intervention group) to cataract surgery alone (329 eyes, control group). Analysis of all data showed that the mean BCVA at 1 week postoperatively had no statistically significant difference in the two groups, but at 1, 3, and 6 months postoperatively, the mean BCVA was statistically significantly better in the anti-VEGF treatment group than that in cataract surgery alone group. Analysis of all data showed that the mean MT was statistically significantly less in the anti-VEGF treatment group at 1 week and 1, 3, and 6 months postoperatively (*P*=0.05, *P*=0.006, *P*=0.0001, and *P*=0.0001, respectively); but postoperative clinical outcomes were differentiated from the type of anti-VEGF agents, IVB or IVR, and the existing macular edema preoperatively. Intravitreal anti-VEGF agent treatment statistically significantly reduced the incidence of diabetic retinopathy progression and maculopathy progression compared to the control group (*P*=0.0003, *P* < 0.00001, respectively).

**Conclusion:**

IVB or IVR treatment immediately after cataract surgery may represent a safe and effective strategy to prevent postoperative macular thickening or reduce macular edema and result in greater mean improvements in visual acuity for diabetic patients.

## 1. Introduction

Diabetic macular edema (DME) is a major cause of vision loss for diabetic patients [1, 2]. Diabetic patients have a higher prevalence of early cataract need for surgery. The evidences revealed that intraocular level of inflammatory cytokines and VEGF in eyes with diabetic retinopathy can be further increased by cataract surgery and significantly increase the risk of DME, which was different from cystoid macular edema (CME) following cataract surgery (Irvine–Gass syndrome) for nondiabetic patients [3–5]. Topical nonsteroidal anti-inflammatory drugs (NSAIDs), either as monotherapy or combined with topical corticosteroids, appear to be effective for the prophylaxis and treatment of CME, but the intravitreal administration of anti-VEGF or steroids, at the moment of cataract surgery, seems to be a feasible solution to prevent or cure diabetic macular edema [6, 7].

Intravitreal anti-VEGF agents are widely accepted as a first-line treatment for use in macular edema following central retinal vein occlusion, wet age-related macular degeneration, diabetic retinopathy, etc. [8–10]. Many studies have reported clinical outcomes of intravitreal bevacizumab (IVB) or intravitreal ranibizumab (IVR) as an adjunct to cataract surgery in the management of diabetic retinopathy (DR) progression, especially macular edema for diabetic patients [11–22]. Due to lack of systematic or larger sample size studies weighting the benefits with the possible adverse effects, the intravitreal anti-VEGF agent treatment remains questionable in diabetic patients undergoing cataract surgery.

In an attempt to detect benefits in safety and efficacy as the primary comparative criteria, we performed a systematic review and meta-analysis of existing randomized controlled trials (RCTs) and high-quality comparative studies of cataract surgery with or without intravitreal anti-VEGF agent treatment for the treatment of DR including nonproliferative diabetic retinopathy (NPDR), proliferative diabetic retinopathy (PDR), or clinically significant macular edema (CSME).

## 2. Materials and Methods

This meta-analysis was performed according to a predetermined protocol described previously [23, 24].

### 2.1. Literature Search

Two reviewers independently searched the following electronic databases: PubMed, EMBASE, and Wanfang Data (e-resources for China studies) and the Cochrane Controlled Trials Register up to June 30, 2018. For maximum sensitivity, we used free text and thesaurus terms including “cataract surgery or phacoemulsification,” “diabetic retinopathy or diabetic macular edema,” and “bevacizumab or Avastin or ranibizumab or Lucentis or aflibercept or anti-vascular endothelial growth factor agents.” All published RCTs and comparative studies comparing cataract surgery alone versus cataract surgery with intravitreal anti-VEGF agent treatment for diabetic retinopathy were included. Patients preparing for cataract surgery were presented with diabetic retinopathy and greater than 18 years. At least one or more clinical outcomes representing clinical outcome parameters must be assessed and published. There was no language restriction on the publications.

The selected studies were appraised by two reviewers, who independently assessed their quality using the methods recommended in the *Cochrane Handbook for Systematic Reviews of Interventions* [25].

Discordance about study inclusion and quality assessment between the two reviewers was resolved through discussion until 100% agreement was reached on the final interpretation of the data.

### 2.2. Outcome Measure

The clinical outcome parameters included mean central macular thickness (CMT), best-corrected visual acuity (BCVA), incidence of diabetic retinopathy and maculopathy progression, incidence of neovascular glaucoma (NVG), and rate of laser photocoagulation treatment postoperatively. Other adverse events such as elevation of intraocular pressure (IOP), retinal detachment, and ocular inflammation were all recorded.

### 2.3. Data Extraction and Analysis

The studies were tabulated and methodologically evaluated to assess homogeneity. Any heterogeneity between the studies would not be justified to pool the assessed outcomes. A customized data extraction form, as described in the Cochrane Handbook for Systematic Reviews of Interventions, was used to record the duration of the trial, sample size, dropouts, the system and ocular baseline features, the inclusion and exclusion criterion for patients, the dosage and location of intravitreal anti-VEGF agent injection, and postoperative treatment.

### 2.4. Statistical Analysis

Quantitative data were analyzed using the Cochrane Review Manager (RevMan) version 5.0 software. Summary estimates, including 95% confidence interval (CI), were calculated. For continuous outcome data (e.g., mean macular thickness) means and standard deviations were used to calculate a weighted mean difference (WMD). For dichotomous outcomes (e.g., incidence of retinopathy progression), the odds ratio (OR) was calculated.

Statistical heterogeneity was tested using Q statistic of chi-square value test and *I*^2^ test. Fixed effects models were used, unless significant evidence of statistical heterogeneity or clinical diversity was found. For results showing significant heterogeneity (*I*^2^ > 50%), random-effects meta-analysis was performed. Outcome measures were assessed on an intent-to-treat (ITT) basis. A *P* value less than 0.05 was considered statistically significant. Subgroup analysis was performed according to the inclusion criterion.

## 3. Results

Nine RCTs and 3 nonrandomized comparative studies published between 2009 and 2016 met the inclusion criteria [11–22]. Each study revealed that there were no significant differences in preoperative demographic features, such as preoperative BCVA and macular thickness that were related to anti-VEGF treatment.

Six studies reported clinical outcomes of intravitreal bevacizumab as an adjunct to cataract surgery in the management of DME [11–15, 21], and 6 studies reported intravitreal ranibizumab management [16–20, 22].

Five studies included NPDR patients with no preexisting DME and evaluated intravitreal anti-VEGF agents at cataract surgery for prevention of postoperative DME [12, 16, 17, 19, 22]; five studies included NPDR patients with preexisting DME and evaluated intravitreal anti-VEGF agents at cataract surgery for management of diabetic maculopathy [13, 14, 18, 20, 21]. Two studies included NPDR or PDR patients with or without CSME and evaluated intravitreal anti-VEGF agents at cataract surgery for management of diabetic maculopathy [11, 15].

Four studies reported that laser photocoagulation was performed according to ETDRS guidelines during the follow-up period [11, 12, 15, 16]. Laser photocoagulation was not performed during the follow-up period in the other 8 studies [13, 14, 17–22].

Additive other treatments, such as intravitreal anti-VEGF agent treatment, were not performed during the follow-up period in the all included studies.

The present meta-analysis involved 338 eyes receiving cataract surgery with anti-VEGF agent treatment and 329 eyes treated with cataract surgery alone. The selection of 12 studies is summarized in Tables 1 and 2.

### 3.1. Macular Thickness

For NPDR without DME, one study [12] applying IVB revealed that the mean MT at 1 month and 6 months postoperatively was statistically significantly less in the IVB treatment group than the control group; 4 studies [16, 17, 19, 22] applying IVR revealed that the MT at 1 week and 1, 3, and 6 months postoperatively was statistically significantly less in the IVR treatment group(Table 3). All data showed that change of postoperative MT from 1 week to 6 months compared with the baseline was minor or increased little in the anti-VEGF treatment group (from −1 to +24 *μ*m), but there was apparent increase of postoperative MT compared with the baseline in the control group (from +12 to +90 *μ*m).

For NPDR with DME, 3 studies [13, 14, 21] applying IVB revealed that the mean MT at 1, 3, and 6 months postoperatively was statistically significantly less in the IVB treatment group than the control group; 2 studies [18, 20] applying IVR revealed that the MT at 1 week and 1, 3, and 6 months postoperatively was statistically significantly less in the IVR treatment group (Table 3). All data showed that change of postoperative MT from 1 week to 6 months compared with the baseline was minor or decreased apparently in the anti-VEGF treatment group (from +10 to −98 *μ*m), but there was apparent increase of postoperative MT compared with the baseline in the control group (from −22 to +77 *μ*m).

Two studies [11, 15] reported the same inclusion criteria: mixed CSME, NPDR, or PDR. And the postoperative laser photocoagulations were performed based on ETDRS criterion. Analysis of these data showed that there was no statistically significant difference of the mean MT in the two groups at 1 and 3 months postoperatively (WMD: −4.34; 95% CI: −39.49, 30.81; *P*=0.81; heterogeneity: *P*=0.92, *I*^2^ = 0%) (WMD: −3.54; 95% CI: −39.45, 32.36; *P*=0.85; heterogeneity: *P*=0.74, *I*^2^ = 0%). However, at 6 months postoperatively, the mean MT was statistically significantly less in the IVB treatment group (WMD: −40.18; 95% CI: −77.01, −3.36; *P*=0.03; heterogeneity: *P*=0.64, *I*^2^ = 0%).

Analysis of all data showed that the mean MT was statistically significantly less in the anti-VEGF treatment group at 1 week and 1, 3, and 6 months postoperatively (Table 3).

### 3.2. BCVA

For NPDR without DME, one study [12] applying IVB revealed that the mean BCVA was statistically significantly better in the IVB treatment group than that in the cataract surgery alone group at 1 month postoperatively, but the mean BCVA at 3 and 6 months postoperatively had no statistically significant difference in the two groups. Three studies [16, 17, 19] applying IVR revealed that the mean BCVA at 1 week postoperatively had no statistically significant difference in the two groups. At 1 and 3 months postoperatively, 1 study [16] reporting LogMAR visual acuity revealed that the mean BCVA had no statistically significant difference in the two groups, and 1 study [19] reporting Snellen visual acuity revealed that the mean BCVA was statistically significantly better in the IVB treatment group. At 6 months postoperatively, the mean BCVA of two studies [16, 17] was statistically significantly better in the IVR treatment group (Table 4).

For NPDR with DME, 3 studies [13, 14, 21] applying IVB revealed that the mean BCVA at 1 week postoperatively had no statistically significant difference in the two groups, but at 1, 3, and 6 months postoperatively, the mean BCVA was statistically significantly better in the IVB treatment group than that in the cataract surgery alone group. Two studies [18, 20] applying IVR revealed that the mean BCVA at 1 week and 1, 3, and 6 months postoperatively was statistically significantly better in the IVR treatment group (Table 4).

Two studies [11, 15] reported the same inclusion criteria: CSME, NPDR, PDR, and previous focal, grid laser photocoagulation for CSME. And the postoperative laser photocoagulation was performed. Analysis of these data showed that the mean BCVA was not statistically significantly different in the both groups at 1, 3, and 6 months postoperatively (WMD: −0.05; 95% CI: −0.24, 0.14; *P*=0.57; heterogeneity: *P*=0.11, *I*^2^ = 60%) (WMD: −0.04; 95% CI: −0.18, 0.10; *P*=0.56; heterogeneity: *P*=0.23; *I*^2^ = 30%) (WMD: −0.02; 95% CI: −0.18, 0.15; *P*=0.82; heterogeneity: *P*=0.11; *I*^2^ = 62%).

Analysis of all data showed that the mean BCVA at 1 week postoperatively had no statistically significant difference in the two groups, but at 1, 3, and 6 months postoperatively, the mean BCVA was statistically significantly better in the anti-VEGF treatment group than that in the cataract surgery alone group (Table 4).

### 3.3. Postoperative Complications and Treatment

The incidence of retinopathy and maculopathy progression after cataract surgery were statistically significantly less in the intravitreal anti-VEGF treatment group (*P*=0.0003, *P* < 0.00001, respectively) than those in the control group (Table 5).

The incidence of NVG progression had no statistically significant difference between both groups, but had a trend to statistical significance (*P*=0.07) (Table 5).

The rate of laser photocoagulation treatment had no statistically significant difference between both groups (*P*=0.72) (Table 5).

There was no significant increase of intraocular pressure after surgery in each group (*P*=0.21) (Table 5).

The incidence of adverse events that were related to the injection itself, such as vitreous hemorrhage and conjunctival hemorrhage, had no statistically significant difference between both groups (*P*=0.97, *P*=0.28, respectively) (Table 5).

No adverse events, such as retinal detachment, severe ocular inflammation, endophthalmitis, or systemic adverse, were reported during the follow-up periods.

## 4. Discussion

The meta-analysis results showed that the intravitreal anti-VEGF agent treatment, irrespective of IVB or IVR, immediately after cataract surgery could prevent the increase in macular thickness of eyes with NPDR without existing DME and decreasing macular thickness in NPDR with existing DME. This effect seemed to hold in 6 months postoperatively.

Similarly, the meta-analysis data also showed the intravitreal anti-VEGF agent treatment could improve better postoperative visual acuity of eyes with NPDR. For NPDR without existing DME, the effect of visual acuity improvement by IVB seemed to hold in one month postoperatively, and the effect of IVR could hold in one to 6 months postoperatively. For NPDR with existing DME, the effect of visual acuity improvement by IVB seemed to hold in 1 to 6 months postoperatively, and the effect of IVR could hold in one week to 6 months postoperatively.

Although there was no study to directly compare IVB to IVR for the treatment of diabetic macular edema immediately after cataract surgery. In the future, more trials will be needed to verify whether IVR was superior to IVB and the therapy effect of IVR was more quickly and more durable for DME after cataract surgery. Many clinical trials compared visual acuity and OCT outcomes associated with IVB vs IVR for the management of DME [26, 27]. IVB and IVR were associated with similar effects on central macular thickness in patients with DME. IVR is associated with greater improvement in BCVA at some study visits, and the mean number of injections is higher in the IVB group.

For subgroups of eyes mixed CSME, NPDR, or PDR with no difference in postoperative macular thickness from 1 month to 3 months, the consequential effect of laser photocoagulation ought to be considered [11, 15]. The reported rate of laser photocoagulation treatment after cataract surgery had no significant difference between the both groups [11, 15]. This reflected that the ocular baseline especially the fundus was similar between both groups because the included studies reported that laser photocoagulation for CSME and PDR was performed promptly after cataract surgery in both groups based on ETDRS criterion. Laser photocoagulation is still a standard therapy for DME and has proven efficacy in large clinical trials [28, 29]. So, the early macular edema or obvious macular edema was treated. Both groups had similar central macular thickness and visual acuity during the 3-month follow-up period. May be the IVB beneficial effect for macular edema was not quick. However, the 6-month data revealed that the IVB treatment still had a little beneficial effect in preventing the macular thickening.

Although the treatment rate of laser photocoagulation had no significant difference, we thought the efficacy of laser photocoagulation after the intravitreous pretreatment group was high. As Lang's study revealed, intravitreal anti-VEGF agent treatment plus laser has also proven to be more effective for the treatment of PDR compared to laser alone [30]. It can improve the resolution of vitreous and retinal hemorrhage and facilitate laser photocoagulation completion. So, the completely laser therapy significantly would reduce the incidence of progression of retinopathy and maculopathy in eyes that received intravitreal anti-VEGF agent treatment. This is a synergistic prophylactic effect determined by laser therapy and intravitreal anti-VEGF agent treatment. Although the incidence of progression of NVG had no significant difference in both groups, the incidence (7/63) of progression of NVG in eyes without receiving intravitreal anti-VEGF agent treatment was more times higher than those (1/62) with receiving treatment, and there was a trend to statistically significant difference.

Many studies have been published to prove whether cataract surgery itself or other factors increase the risk of DR progression postoperatively [31, 32]. Whatever reasons, cataract surgery must be performed for some diabetic patients. And how to prevent postoperatively macular thickening and retinopathy progression is the main question. Besides the beneficial effect of intravitreal anti-VEGF agent treatment, the traditional treatment still yielded better results in terms of macular thickness reduction and improvement of VA, such as laser photocoagulation, intravitreal triamcinolone, or dexamethasone implant treatment [7, 33–35], but intravitreal triamcinolone treatment was associated with a greater risk of intraocular pressure elevation. An optional therapy was determined by not only the better clinical outcomes but also the fewer side effects. The selected trials revealed that intravitreal anti-VEGF agent treatment was well tolerated, and no local and systemic adverse events were noticed during the study. However, from the values of macular thickness during follow-up, there was a trend of macular thickening from 3 to 6 months postoperatively [15, 16]. The drawback of its nonpermanent effect and higher costs ought to be of concern [36]. Further therapy for diabetic retinopathy after cataract surgery was integrated by different treatment methods according to their respective advantages.

This present meta-analysis might have some limitations. One weakness of our study was that there was no trial exclusively focused on PDR as subgroup analysis. The meta-analysis results should be accurate and comprehensive if adding the subgroup analysis of PDR with or without macular edema. Secondly, the studies included were heterogeneous in terms of study location, population, number of patients from different studies and basal condition, and study quality (relative methodological strengths and weaknesses). The strengths of this study include the randomized study design and nonrandomized comparative study design. The baseline conditions in these nonrandomized comparative studies were highly controlled. So it is feasible to include these studies to perform meta-analysis. More study participants were included to research, and the results were more closed to be representative of the real-world population with DME. These findings still need to be interpreted with caution. Bevacizumab is an off-label anti-VEGF agent for DME or postsurgical macular edema. It must be used with caution.

In conclusion, the present study demonstrated that intravitreal anti-VEGF agent treatment plus cataract surgery may represent a safe and effective strategy to prevent postoperative macular thickening or decreasing macular edema and result in greater mean improvements in visual acuity for diabetic patients. In future, new treatments, such as other anti-VEGF agent injection and intravitreal anti-VEGF agents combined subtenon triamcinolone injection, should be investigated to improve clinical efficacy, and high-quality, large-scale, multicenter randomized control trials will be needed to verify [37]. The optimized therapy should be suggested when using the data from the evidence-based study to guide treatment considerations for an individual patient.

## Figures and Tables

**Table 1:** Characteristics of studies of intravitreal anti-VEGF agent treatment after cataract surgery versus cataract surgery alone included in the meta-analysis.

Trials	Location	Design of trial	Duration (m)	Original patients (eyes)	Dropouts	Mean age ± SD (range) (M/F)	System baselines	Ocular baselines
Salehi et al. [11]	Iran	Prospective, randomized controlled trial	6	(1): 27 (27) (2): 30 (30)	(1): 0 (0) (2): 0 (0)	(1): 61.5 ± 12.7 (14/13) (2): 62.3 ± 14 (17/13)	NS: DM type and duration, hypertension duration	NS: BCVA, MT
Fard et al. [12]	Iran	Prospective, randomized controlled trial	6	(1): 31 (31) (2): 32 (32)	(1): 0 (0) (2): 2 (2)	(1): 64 ± 4 (13/18) (2): 62 ± 5 (15/15)	NS: DM type and duration, coronary artery disease percentage, HbA1c level, hypertension duration	NS: BCVA, MT
Takamura et al. [13]	Japan	Prospective, randomized, masked cohort study.	3	(1): 21 (21) (2): 21 (21)	(1): 0 (0) (2): 0 (0)	(1): 67.3 ± 5.2 (9/12) (2): 69.1 ± 5.9 (10/11)	NS: DM duration, HbA1c level	NS: cataract severity (LOCS), BCVA, RT
Lanzagorta-Aresti et al. [14]	Spain	Prospective, randomized pilot study	6	(1): 13 (13) (2): 13 (13)	(1): 0 (0) (2): 0 (0)	NS: age and sex.	NR	NS: BCVA, MT
Cheema et al. [15]	Kingdom of Saudi Arabia	Prospective, randomized controlled trial	6	(1): 35 (35) (2): 33 (33)	(1): 0 (0) (2): 0 (0)	(1): 66.14 (22/13) (2): 64.5 (21/12)	NS: DM type and duration, hypertension duration	NS: retinopathy and maculopathy type, BCVA, MT
Chae et al. [16]	Korea	Prospective randomized study	6	(1): 40 (40) (2): 40 (40)	(1): 1 (1) (2): 3 (3)	(1): 62.9 (21/18) (2): 67.2 (20/17)	NS: DM type	NS: BCVA, CST, TMV, PRP history
Lin et al. [17]	China	Prospective, randomized controlled trial	6	(1): 23 (23) (2): 23 (23)	(1): 0 (0) (2): 0 (0)	(1): 64.6 (8/15) (2): 65.5 (10/13)	NR	NS: BCVA, MT
Lu et al. [18]	China	Nonrandomized comparative study	6	(1): 17 (19) (2): 18 (19)	(1): 0 (0) (2): 0 (0)	(1): 59.59 (2): 57.56	NR	NS: BCVA, MT, IOP
Cheng et al. [19]	China	Nonrandomized comparative study	6	(1): 43 (64) (2): 43 (63)	(1): 0 (0) (2): 0 (0)	(1): 43.3 (20/23) (2): 42.7 (19/24)	NR	NS: BCVA, MT
Li [20]	China	Prospective, randomized controlled trial	6	(1): 20 (24) (2): 18 (19)	(1): 0 (0) (2): 0 (0)	(1): 59.6 (2): 59.1	NS: DM type	NS: BCVA, MT, IOP
Chen et al. [21]	Taiwan	Retrospective comparative study	3	(1): 14 (15) (2): 14 (14)	(1): 0 (0) (2): 0 (0)	(1): 66 (6/8) (2): 65 (7/7)	NS: age, sex, eye laterality	NS: BCVA, MT
Udaondo et al. [22]	Spain	Prospective, randomized controlled trial	3	(1): 27 (27) (2): 27 (27)	(1): 0 (0) (2): 0 (0)	(1): 68.9 ± 4.7 (10/17) (2): 72.8 ± 5.2 (9/18)	NS: age, sex	NS: MT

VEGF = vascular endothelial growth factor; (1) = intravitreal anti-VEGF agent treatment after cataract surgery group; (2) = cataract surgery alone group; SD = standard deviations; m = month; M = male; F = female; DM = diabetes mellitus; HbA1C = glycosylated hemoglobin A1C; BCVA = best-corrected visual acuity; MT = macular thickness; NS = not significant; NR = not reported; LOCS = the Lens Opacities Classification System III.

**Table 2:** Characteristics of surgical procedures of IVB or IVR treatment after cataract surgery versus cataract surgery alone included in the meta-analysis.

Trial	Inclusion	Exclusion	Dosage	IVB location	Laser photocoagulation during follow-up	Surgeon
Salehi et al. [11]	(1) CSME; (2) mild, moderate, severe, or very severe NPDR or PDR; or (3) a combination of 1 and 2. Patients with previous focal or grid laser photocoagulation for CSME were eligible	Those with previous PRP for PDR	IVB: 1.25 mg	3.5 mm posterior to the inferotemporal limbus using a 27-gauge needle	Laser photocoagulation was performed according to ETDRS guidelines	One
Fard et al. [12]	Moderate or severe NPDR, preoperative visual acuity ≤20/50, preoperative central MT < 200 *μ*m	Prior laser photocoagulation in the study eye	IVB: 1.25 mg	3.5 mm posterior to limbus with a 30-gauge needle	PRP was performed according to ETDRS guidelines for DR	One
Takamura et al. [13]	NPDR with DME (MT > 300 *μ*m), DME had occurred 3 to 18 months earlier, DME involved the fovea, and BCVA was ≤20/40	PDR were excluded. No patients had undergone photocoagulation within the previous 12 months	IVB: 1.25 mg	30-gauge needle	No	NR
Lanzagorta-Aresti et al. [14]	Moderate NPDR with diffuse macular edema, lasered with macular grid at 2 and 3 months preoperatively, central MT > 200 *μ*m	Other associated ocular diseases capable of causing macular edema, patients who had had suffered complications during surgery or in the postoperative period	IVB: 1.25 mg	3.5 mm from the limbus via a 30-gauge needle	No	Same
Cheema et al. [15]	(1) CSME; (2) mild, moderate, severe, or very severe NPDR or PDR; or (3) a combination of 1 and 2. Patients with previous focal or grid laser photocoagulation for CSME were eligible	Those with previous PRP for PDR	IVB: 1.25 mg	3.0 to 3.5 mm from the limbus with a 27-gauge needle	Laser photocoagulation was performed according to ETDRS guidelines	Same
Chae et al. [16]	(1) Patients with NPDR, or patients with stable DR, who had completed PRP at least 3 months earlier; (2) patients with visually significant cataract with BCVA under 20/30; (3) patients with central subfield thickness (CST) that was <300 *μ*m	Any kind of intravitreal drug injection within the previous 3 months; retinal laser treatment of diabetic ME within the previous 3 months, conditions (e.g., chronic ME, anatomical macular problem, and severe macular infarction) that the investigators believed are associated with a low probability of visual acuity restoration	IVR: 0.5 mg	3 mm posterior to the limbus	Additive PRP due to vitreous hemorrhage	Four surgeons
Lin et al. [17]	NPDR without clinical DME	PDR was excluded, any kind of intravitreal drug injection preoperatively	IVR: 0.5 mg	NR	No	One
Lu et al. [18]	NPDR with clinical DME		IVR: 0.5 mg	3.5 mm posterior to the inferotemporal limbus	No	One
Cheng et al. [19]	NPDR without clinical DME		IVR: 0.5 mg		No	NR
Li [20]	NPDR with clinical DME	PDR, AMD, high myopia, intraocular surgery history	IVR: 0.5 mg	NR	No	One
Chen et al. [21]	Diffuse central macular edema of at least 250 mm, as demonstrated by OCT	Received laser photocoagulation less than 3 months before enrollment; concurrent PDR; IV injection of drugs within 6 months	IVB: 2.5 mg	3.5 mm posterior to the inferotemporal limbus		
Udaondo et al. [22]	Mild to moderate NPDR without clinical DME	Previous DME treated or not, any kind of complication during the surgery, other ocular pathology with macular involvement	IVR: 0.5 mg	NR	No	Same

IVB = intravitreal bevacizumab; IVR = intravitreal ranibizumab; PDR = proliferative diabetic retinopathy; NPDR = nonproliferative diabetic retinopathy; MT = macular thickness; PRP = panretinal photocoagulation; NR = not reported; DR = diabetic retinopathy; CSME = the presence of clinically significant macular edema; BCVA = best-corrected visual acuity; DME = diabetic macular edema; ETDRS = the early treatment diabetic retinopathy study.

**Table 3:** Meta-analytic findings about postoperative macular thickness compared to the baseline.

	No. of studies	Mean difference (95% CI)	*P*	*I* ^2^ (%)	*P* for heterogeneity
MT after 1 week					
IVB for NPDR without DME	—				
IVR for NPDR without DME	2 [16, 19]	−11.69 (−19.56, −3.83)	0.004	0	0.80
IVB for NPDR with DME	—				
IVR for NPDR with DME	1 [18]	−125.07 (−156.34, −93.80)	0.00001	Not applicable	
Total	3 [16, 18, 19]	−45.95 (−91.49, −0.41)	0.05	96	0.00001

MT after 1 month					
IVB for NPDR without DME	1 [12]	−90 (−105.11, −74.89)	0.00001	Not applicable	
IVR for NPDR without DME	3 [16, 19, 22]	−16.56 (−26.10, −9.02)	0.0001	8	0.34
IVB for NPDR with DME	2 [13, 21]	−84.89 (−147.88, −21.91)	0.008	63	0.10
IVR for NPDR with DME	1 [18]	−153.83 (−212.59, −95.07)	0.00001	Not applicable	
Total	9 [11–13, 15, 16, 18, 19, 21, 22]	−49.92 (−78.34, −21.51)	0.0006	93	0.00001

MT after 3 months					
IVB for NPDR without DME	—				
IVR for NPDR without DME	3 [16, 19, 22]	−25.64 (−32.54, −18.74)	0.00001	3	0.36
IVB for NPDR with DME	3 [13, 14, 21]	−53.55 (−72.29, −34.80)	0.00001	0	0.57
IVR for NPDR with DME	1 [18]	−108.80 (−170.64, −46.96)	0.0006	Not applicable	
Total	9 [11, 13–16, 18, 19, 21, 22]	−34.73 (−49.94, −19.51)	0.00001	58	0.01

MT after 6 months					
IVB for NPDR without DME	1 [12]	−24.00 (−34.50, −13.50)	0.00001	Not applicable	
IVR for NPDR without DME	2 [16, 17]	−24.77 (−43.24, −6.30)	0.009	0	0.88
IVB for NPDR with DME	1 [14]	−109.84 (−174.48, −45.20)	0.0009	Not applicable	
IVR for NPDR with DME	2 [18, 20]	−90.25 (−127.24, −53.26)	0.00001	0	0.58
Total	8 [11, 12, 14–18, 20]	−45.22 (−65.37, −25.07)	0.00001	62	0.01

IVB = intravitreal bevacizumab; IVR = intravitreal ranibizumab; NPDR = nonproliferative diabetic retinopathy; MT = macular thickness; DME = diabetic macular edema.

**Table 4:** Meta-analytic findings about postoperative best-corrected visual acuity compared to the baseline.

	No. of studies	Mean difference (95% CI)	*P*	*I* ^2^ (%)	*P* for heterogeneity
BCVA after 1 week					
IVR for NPDR without DME (LogMAR)	1 [16]	−0.06 (−0.18, 0.06)	0.32	Not applicable	
IVR for NPDR without DME (Snellen)	2 [17, 19]	0.04 (−0.04, 0.12)	0.35	50	0.16
IVB for NPDR with DME (LogMAR)	1 [21]	−0.13 (−0.51, 0.25)	0.50	Not applicable	
IVR for NPDR with DME (Snellen)	1 [18]	0.16 (0.02, 0.30)	0.02	Not applicable	
Total: (LogMAR)	2 [16, 21]	−0.07 (−0.18, 0.05)	0.25	0	0.73
Total: (Snellen)	3 [17–19]	0.06 (−0.01, 0.14)	0.11	51	0.13

BCVA after 1 month					
IVB for NPDR without DME (LogMAR)	1 [12]	−0.24 (−0.34, −0.14)	0.00001	Not applicable	
IVR for NPDR without DME (LogMAR)	1 [16]	−0.07 (−0.17, 0.03)	0.15	Not applicable	
IVR for NPDR without DME (Snellen)	1 [19]	0.15 (0.09, 0.21)	0.00001	Not applicable	
IVB for NPDR with DME (LogMAR)	2 [13, 21]	−0.20 (−0.37, −0.03)	0.02	0	0.75
IVR for NPDR with DME (Snellen)	1 [18]	0.19 (0.01, 0.37)	0.04	Not applicable	
Total: (LogMAR)	6 [11–13, 15, 16, 21]	−0.14 (−0.19, −0.09)	0.0001	50	0.07
Total: (Snellen)	2 [18, 19]	0.15 (0.10, 0.21)	0.00001	0	0.68

BCVA after 3 months					
IVB for NPDR without DME (LogMAR)	1 [12]	−0.04 (−0.14, 0.06)	0.41	Not applicable	
IVR for NPDR without DME (LogMAR)	1 [16]	−0.03 (−0.12, 0.06)	0.50	Not applicable	
IVR for NPDR without DME (Snellen)	1 [19]	0.20 (0.15, 0.25)	0.00001	Not applicable	
IVB for NPDR with DME (LogMAR)	2 [13, 21]	−0.13 (−0.24, −0.02)	0.02	0	0.91
IVB for NPDR with DME (Snellen)	1 [14]	0.19 (0.02, 0.36)	0.03	Not applicable	
IVR for NPDR with DME (Snellen)	1 [18]	0.23 (0.05, 0.41)	0.01	Not applicable	
Total: (LogMAR)	6 [11–13, 15, 16, 21]	−0.06 (−0.11, −0.01)	0.02	0	0.58
Total: (Snellen)	3 [14, 18, 19]	0.20 (0.16, 0.25)	0.00001	0	0.94

BCVA after 6 months					
IVB for NPDR without DME (LogMAR)	1 [12]	0.00 (−0.10, 0.10)	1.00	Not applicable	
IVR for NPDR without DME (LogMAR)	1 [16]	−0.13 (−0.24, −0.02)	0.02	Not applicable	
IVR for NPDR without DME (Snellen)	1 [17]	0.15 (0.05, 0.25)	0.005	Not applicable	
IVB for NPDR with DME (Snellen)	1 [14]	0.26 (0.10, 0.42)	0.002	Not applicable	
IVR for NPDR with DME (Snellen)	2 [18, 20]	0.19 (0.10, 0.28)	0.00001	0	1.00
Total: (LogMAR)	4 [11, 12, 15, 16]	−0.07 (−0.11, −0.02)	0.006	21	0.29
Total: (Snellen)	4 [14, 17, 18, 20]	0.19 (0.12, 0.25)	0.00001	0	0.74

IVB = intravitreal bevacizumab; IVR = intravitreal ranibizumab; NPDR = nonproliferative diabetic retinopathy; BCVA = best-corrected visual acuity; DME = diabetic macular edema.

**Table 5:** Postoperative events and treatment in the meta-analysis.

Events	No. of studies	Crude rate, *n*/*N*		Rate difference % (95% CI)	*P* for overall effect	*I* ^*2*^ for heterogeneity (%)	*P* for heterogeneity
		Intervention	Control				
DR progression	3 [1, 2, 5]	12/93	34/93	0.26 [0.13, 0.54]	0.0003	27	0.26
Maculopathy progression	3 [1, 5, 6, 12]	18/128	68/127	0.10 [0.05, 0.20]	<0.00001	0	0.81
NVG progression	2 [1, 5]	1/62	7/63	0.19 [0.03, 1.13]	0.07	0	0.97
Laser photocoagulation	4 [1, 2, 5, 6]	36/132	34/130	1.13 [0.59, 2.13]	0.72	0	0.86
VH	1 [6]	1/39	1/37	0.95 [0.06, 15.72]	0.97	—	—
Elevated IOP	2 [7, 9]	3/87	0/86	4.12 [0.45, 37.82]	0.21	0	0.83
CH	1 [7]	2/23	0/23	5.47 [0.25, 120.37]	0.28	—	—

IVB = intravitreal bevacizumab; DR = diabetic retinopathy; NVG = neovascular glaucoma; CI = confidence interval; VH = vitreous hemorrhage; CH = conjunctival hemorrhage.
